# AKR1C3 Is Associated with Better Survival of Patients with Endometrial Carcinomas

**DOI:** 10.3390/jcm9124105

**Published:** 2020-12-19

**Authors:** Marko Hojnik, Nataša Kenda Šuster, Špela Smrkolj, Snježana Frković Grazio, Ivan Verdenik, Tea Lanišnik Rižner

**Affiliations:** 1 Institute of Biochemistry, Faculty of Medicine, University of Ljubljana, 1000 Ljubljana, Slovenia; marko.hojnik@mf.uni-lj.si; 2 Division of Gynecology, Department of Obstetrics and Gynecology, University Medical Centre Ljubljana, 1000 Ljubljana, Slovenia; natasa@kenda.si (N.K.Š.); spela.smrkolj@mf.uni-lj.si (Š.S.); ivan.verdenik@guest.arnes.si (I.V.); 3 Medical Faculty, University of Ljubljana, 1000 Ljubljana, Slovenia; 4 Division of Gynecology, Department of Pathology, University Medical Centre Ljubljana, 1000 Ljubljana, Slovenia; snjezana.frkovicgrazio@kclj.si

**Keywords:** endometrial carcinoma, high grade serous ovarian carcinoma, prognosis, immunohistochemistry, biomarker, aldo-keto reductase 1C3 (AKR1C3)

## Abstract

The aldo-keto reductase (AKR) superfamily is gaining attention in cancer research. AKRs are involved in important biochemical processes and have crucial roles in carcinogenesis and chemoresistance. The enzyme AKR1C3 has many functions, which include production of prostaglandins, androgens and estrogens, and metabolism of different chemotherapeutics; AKR1C3 is thus implicated in the pathophysiology of different cancers. Endometrial and ovarian cancers represent the majority of gynecological malignancies in developed countries. Personalized treatments for these cancers depend on identification of prognostic and predictive biomarkers that allow stratification of patients. In this study, we evaluated the immunohistochemical (IHC) staining of AKR1C3 in 123 paraffin-embedded samples of endometrial cancer and 99 samples of ovarian cancer, and examined possible correlations between expression of AKR1C3 and other clinicopathological data. The IHC expression of AKR1C3 was higher in endometrial cancer compared to ovarian cancer. In endometrioid endometrial carcinoma, high AKR1C3 IHC expression correlated with better overall survival (hazard ratio, 0.19; 95% confidence interval, 0.06−0.65, *p* = 0.008) and with disease-free survival (hazard ratio, 0.328; 95% confidence interval, 0.12–0.88, *p* = 0.027). In patients with ovarian cancer, there was no correlation between AKR1C3 IHC expression and overall and disease-free survival or response to chemotherapy. These results demonstrate that AKR1C3 is a potential prognostic biomarker for endometrioid endometrial cancer.

## 1. Introduction

### 1.1. Endometrial Cancer

The majority of gynecological cancers in the more developed countries are endometrioid endometrial cancer and high grade serous ovarian cancer (HGSC). Endometrial cancer is the second most common carcinoma of the female genital tract globally, after cervical cancer, and it represents a major public health problem [1,2]. In 2018, 382,069 new cases and 89,929 deaths were estimated worldwide, according to Globocan [2]. In Slovenia, the estimated incidence of endometrial cancer for 2020 is 38 cases per 100,000 women with a slightly increasing trend over the last 50 years (average annual increase, 2.8%) [3].

Endometrial carcinoma can be divided into two subgroups: type 1 (80%), which includes estrogen-dependent carcinomas (e.g., endometrioid and mucinous carcinomas); and type 2, which includes estrogen-independent and more aggressive carcinomas (mainly serous carcinomas). Recent data have suggested that estrogens might also have roles in these type 2 cancers [4]. According to molecular classification, there are four groups of endometrial cancers: polymerase epsilon (POLE) ultramutated; Microsatellite instability (MSI) hypermutated; copy-number-low/p53-wild-type (p53wt); and copy-number-high/p53-mutated (p53mt) [5]. The most relevant prognostic factor for endometrial cancer is the disease stage, which can only be determined after radical surgery. New biomarkers are thus needed to predict pathological behaviors of different cancers on biopsy or curettage, to allow stratification of patients before surgery.

### 1.2. Ovarian Cancer

Ovarian cancer is the sixth leading cause of cancer-related deaths in Europe. In 2018, 295,414 new cases and 184,799 deaths were estimated worldwide, according to Globocan [2]. In Slovenia, the estimated incidence of ovarian cancer is 13 cases per 100,000 women for 2020, with a slightly decreasing trend over the last few years. Most patients are diagnosed with advanced-stage disease; they thus have poor prognosis, with 5-year overall survival (OS) of only 40.0% [3].

Ovarian cancer is a heterogeneous disease that includes several types of tumors with large differences in their histopathological features and clinical behaviors [6]. The most prevalent histological type of ovarian carcinoma is HGSC. According to molecular classification, there are four subtypes of HGSC [7]: immunoreactive, differentiated, proliferative, and mesenchymal. HGSC is a very aggressive tumor with high mortality rates, and is also usually discovered in an advanced stage of disease (75–80% of cases). Virtually all patients with HGSC receive cytotoxic chemotherapy. There are relatively few biomarkers of prognosis in HGSC, and none of them have been introduced into routine clinical practice.

### 1.3. Biomarkers for Endometrial and Ovarian Cancers

There is a clear need for new methods for early diagnosis and prognosis of endometrial and ovarian cancers. A few studies in endometrial cancer and a large number of studies in ovarian cancer have focused on identification of diagnostic, prognostic, and predictive biomarkers [8,9]. Potential biomarkers for endometrial and ovarian cancers include proteins involved in carcinogenesis as well as proteins involved in chemoresistance.

### 1.4. Aldo-Keto Reductase 1C3

The enzymes of the aldo-keto reductase (AKR) superfamily have roles in a plethora of important biochemical processes, including chemoresistance. AKR1C3 catalyzes the reduction of prostaglandin (PG)H2 to PGF2α, and PGD2 to 9α,11β-PGF2, both of which indirectly activate mitogen-activated protein kinase (MAPK) and inhibit peroxisome proliferator-activated receptor (PPAR)γ, which can lead to cell proliferation [10,11,12]. AKR1C3 also has roles in the metabolism of steroid hormones, as it catalyzes the reduction of androstenedione to testosterone, and estrone to estradiol, which can stimulate cancer cell proliferation [13,14,15,16]. In this manner, AKR1C3 can be involved in the pathogenesis of endometrial and ovarian cancers. However, the pathophysiological role of this enzyme depends on the availability of substrates and coenzymes within the cancerous cells.

AKR1C enzymes have previously been associated with chemoresistance in ovarian cancer, as several microarray and qPCR studies have reported up-regulation of AKR1C genes in ovarian cancer tissue, as well as in cisplatin-resistant, doxorubicin-resistant, and even in dual carboplatin/docetaxel-resistant cell lines [17,18]. The AKR1C enzymes have been shown to regulate the generation of cisplatin-associated reactive oxygen species, and to disrupt apoptotic pathways [19]. AKR1C3 also has an important role in the metabolism of chemotherapeutics, such as anthracyclines, doxorubicin, vincristine, and daunorubicin [20,21,22]. The involvement of the AKR1Cs in chemoresistance thus includes inactivation of cellular stress caused by either chemotherapeutics, inactivation of chemotherapeutics, or both [19].

The aim of this study was to examine expression of AKR1C3 in endometrial and ovarian cancers, and possible correlations between AKR1C3 expression and clinicopathological data, along with evaluation of AKR1C3 as a prognostic biomarker of OS and a predictive biomarker for chemotherapy response.

## 2. Materials and Methods

### 2.1. Study Groups

This retrospective study included 123 patients with endometrial cancer (selected cases were diagnosed from 2003 to 2014), as 104 with type I endometrial cancer and 19 with type II endometrial cancer, plus 99 patients with HGSC (selected cases were diagnosed from 2002 to 2012). Only samples of the primary tumors were included in this study. Demographic and histopathological data of these cases with endometrial and ovarian cancers are given in Table 1 and Table 2, respectively.

We first evaluated immunohistochemical (IHC) staining of AKR1C3 in the 123 samples of endometrial cancer and the 99 samples of HGSC. For endometrial cancer, we compared the results with adjacent nonneoplastic endometrial tissue from the same patients, when this tissue was available. We then examined correlations with the other clinicopathological data, including time to progression, survival, stage of disease, lymphovascular invasion, response to chemotherapy, menopausal status, parous status, smoking, and use of hormone replacement therapy.

### 2.2. Immunohistochemistry

Immunohistochemical staining was performed on formalin-fixed, paraffin-embedded tissue samples of endometrial and ovarian cancer. All of the samples were obtained from the archive of the Division of Gynecology, Department of Pathology, University Medical Centre Ljubljana, and were re-evaluated before inclusion in this study.

Tissue slides of 3–5 µm thickness were cut from each paraffin-embedded tissue block and placed on glass slides (Superfrost Plus; Thermo Scientific, Leicestershire, UK). The slides were dried in a ventilation slide-drying oven for 60 min at 60 °C. IHC staining for AKR1C3 was carried out on an automatic system (BenchMark GX, Ventana, Basel, Switzerland) using detection kits (OptiView DAB; Ventana, Basel, Switzerland; cat. no. 760-700), according to the manufacturer’s instructions.

**Figure 1:**
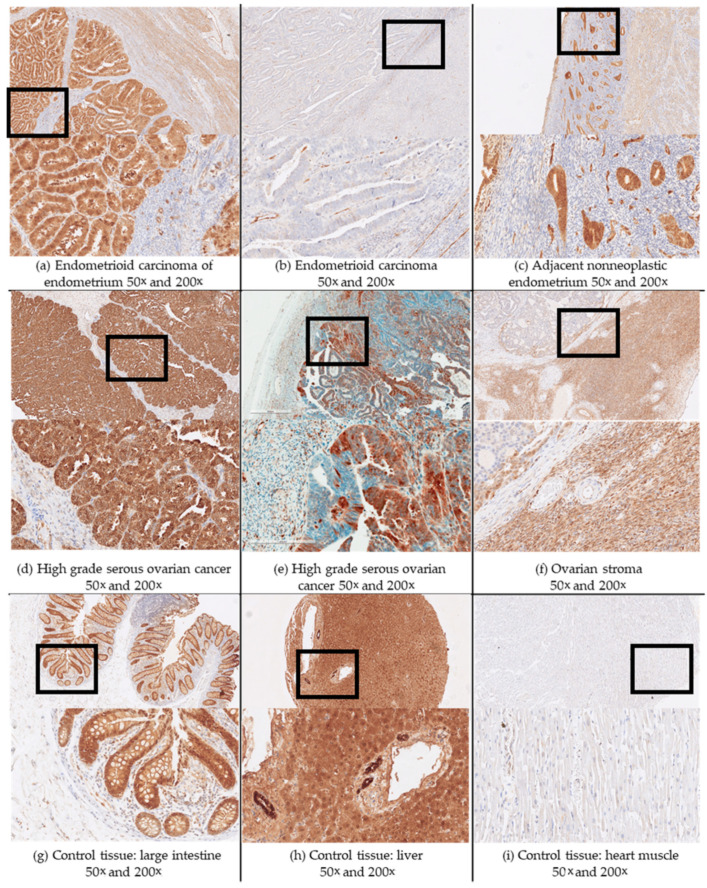
Representative immunohistochemistry staining for AKR1C3 in samples of endometrial and ovarian cancers, and in control tissue. Upper half of panels, 50× magnification. Black box fields, area shown below at 200× magnification.

Tissue sections were deparaffinized (EZPrep solution cat. no. 950-102, Ventana, Basel, Switzerland) for 4 min at 72 °C. Epitope retrieval was achieved using a Tris-based buffer, pH 8.5, for 24 min, at 95 °C (cell conditioning solution CC1 cat. no. 950-124, Ventana, Basel, Switzerland). The slides were incubated with the primary antibodies (anti-AKR1C3; Sigma-Aldrich; cat. no. A6229 NP6.G6.A6) for 30 min (optimized dilution in Tris-buffered saline, 1:400). The positive controls for AKR1C3 were colonic mucosa (epithelium of the crypts) and normal liver tissue (hepatocytes), and the negative controls were lung parenchyma and cardiomyocytes (Figure 1; Supplementary Materials, Table S3). The evaluation was based on the proportions (%) of stained cells. The stained tissue sections were evaluated by two independent observers (authors S.F.G. and M.H.). Inter-observer reproducibility was determined by the interclass correlation coefficient, which was >0.9 for both endometrial and ovarian cancers.

### 2.3. Statistics

Student’s t-tests were used to compare mean expression (as percentage of positive cells) between endometrial cancer and adjacent nonneoplastic endometrium. Linear regression analysis was performed to evaluate correlations between AKR1C3 IHC expression and weight, height, and body mass index. Kaplan–Meier analysis and Cox regression analysis were performed to evaluate survival. Mann–Whitney U and Kruskal–Wallis tests were used to test for correlations between AKR1C3 IHC expression and the other clinical data. All of the tests were performed using SPSS software (IBM version 22, Armonk, NY, USA).

### 2.4. Ethical Issues

This study was retrospective and was approved by the Republic of Slovenia National Medical Ethics Committee (0120-701/2017-6).

## 3. Results

### 3.1. Demographic and Histopathological Characteristics of Patients with Endometrial and Ovarian Cancers

The endometrial tumor cohort (*n* = 123) included 104 endometrioid (majority, grade I), and 19 type 2 endometrial cancers (12 serous, 3 dedifferentiated, 1 clear cell, 1 mixed carcinoma, 2 carcinosarcomas). The majority of the patients (*n* = 103 cases) were diagnosed with FIGO stage I disease. The 5-year OS was 87%. Follow-up was available for 123 out of 123, and ranged from 0.4 to 17.6 years (median, 7.6 years).

The ovarian cancers included 99 HGSC cases. The majority of these patients (*n* = 80) were diagnosed with advanced FIGO stage III or IV disease. Follow-up was available for 97 out of 99, and ranged from 3.6 months to 11.3 years (median, 37.2 months). In 53 out of the 71 patients with follow-up data who received chemotherapy, disease-free survival (DFS) of at least six months was achieved, and this group was defined as responders. The other group of patients where DFS was not achieved by six months was defined as nonresponders. The 5-year OS was 22.2% for nonresponders, compared to 28.3% for responders. All of the collected clinical data are given in Table 1 and Table 2.

### 3.2. AKR1C3 Expression Levels in Endometrial Cancer and Their Correlation with Survival and Other Clinical Data

**Figure 2:**
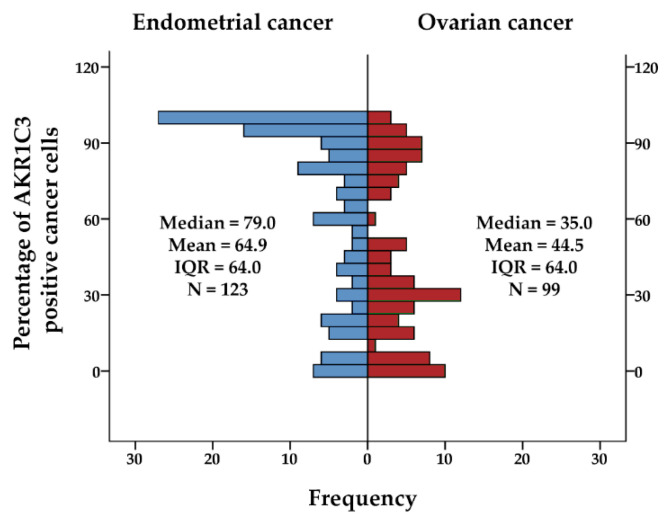
AKR1C3 immunohistochemical staining as percentage of positive cancer cells of the paraffin sections of the endometrial cancers and ovarian cancers; IQR, interquartile range; *N*, number of patients.

For endometrial cancer, positive IHC reactions were seen for AKR1C3 in the cytoplasm and also nucleus of the epithelial cancer cells in endometrioid and nonendometrioid endometrial cancers, including serous, dedifferentiated, carcinosarcomas, clear cell, and mixed carcinoma. The IHC reaction was also seen for endothelium and myometrium (Figure 1; Supplementary Materials, Figure S1). The median and mean percentage of AKR1C3 positive cancer cells were 78.5 and 64.6 for endometrioid cancers, and 79.0 and 66.7 for nonendometrioid cancers (Figure 2). Adjacent nonneoplastic endometrium showed strong positive reactions in the cytoplasm and also the nucleus in endometrial glands, with median percentage of AKR1C3 positive epithelial cells 95.0 and mean 89.0; endothelium, myometrium, and endometrial stroma were negative (Figure 1).

**Figure 3:**
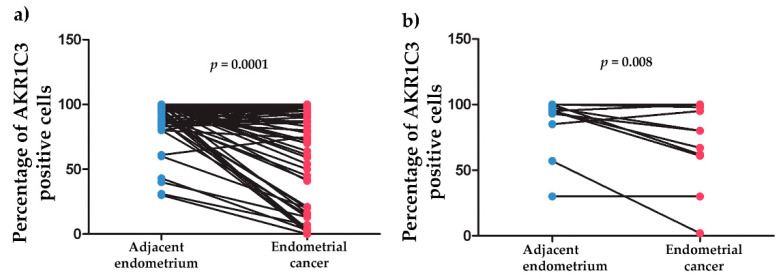
AKR1C3 immunohistochemistry expression in endometrial cancers and the surrounding nonneoplastic endometrium. (**a**) Surrounding nonneoplastic endometrium (*N* = 61, mean = 89.5, median = 95.0, IQR = 13.8) compared to endometrioid endometrial cancer (*N* = 61, mean 66.2, median 83.0, IQR = 67.0). (**b**) Surrounding nonneoplastic endometrium (*N* = 11, mean = 86.1, median = 95.0, IQR = 15.0) compared to nonendometrioid endometrial cancer (*N* = 11, mean = 70.4, median = 80.0, IQR = 37.0); IQR, interquartile range; N, number of patients.

Only 72 of the 123 cases of endometrial carcinoma included some nonneoplastic endometrial tissue adjacent to the carcinoma that was available for IHC analysis. Significantly higher percentages of positive epithelial cells were seen for adjacent nonneoplastic endometrium (89.5 ± 2.2) compared to endometrioid endometrial cancer (66.2 ± 4.7) (*p* = 1 × 10^−4^) (Figure 3a). In the group of nonendometrioid endometrial cancers, there were also significant differences between adjacent nonneoplastic endometrium (86.1 ± 6.7) and cancer cells (70.4 ± 9.4) (*p* = 0.008) (Figure 3b).

**Figure 4:**
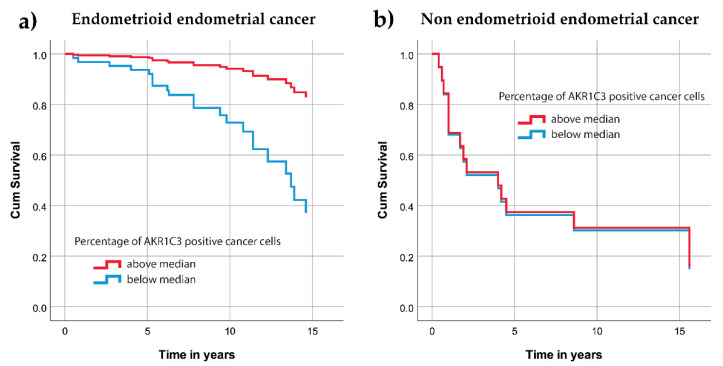
Cumulative survival curves for the AKR1C3 groups separated according to the median values. (**a**) Patients with endometrioid endometrial cancer. (**b**) Patients with nonendometrioid endometrial cancer.

In further analysis, endometrioid endometrial cancer was evaluated separately from nonendometrioid types of endometrial cancer. The samples were first divided into quintiles according to their percentage of AKR1C3 positive cancer cells, with the following border values: 21, 60, 89, and 98. Due to a lack of cases in one of the quintile groups, the median AKR1C3 expression was used as the border value (79%) to divide the cases into two groups. Then Cox survival models were computed. As shown by the survival curves (Figure 4a), cases with >79% of positive cells were differentiated from the other cases. For this group of patients the risk of death was significantly lower (hazard ratio, 0.19; 95% confidence interval, 0.06–0.65, *p* = 0.008). When nonendometrioid endometrial cancers were analyzed, there were no significant differences for survival (Figure 4b).

**Figure 5:**
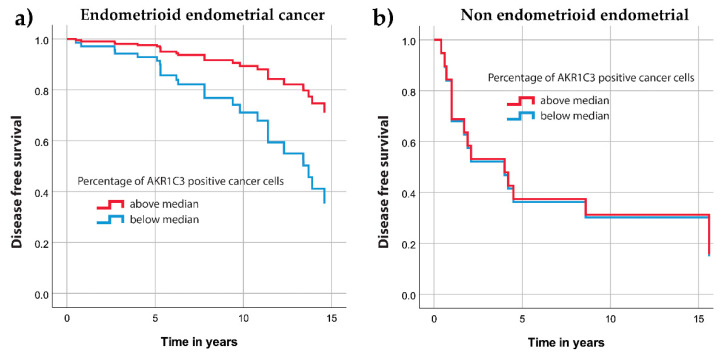
Disease-free survival curves for the AKR1C3 groups separated according to the median value. (**a**) Patients with endometrioid endometrial cancer. (**b**) Patients with nonendometrioid endometrial cancer.

Similarly, Cox regression analysis revealed significantly longer disease free survival for endometrioid endometrial cancer patients with above median percentage of AKR1C3 positive cells (hazard ratio, 0.328, 95% confidence interval, 0.12–0.88, *p* = 0.027) (Figure 5a) while for nonendometrioid endometrial cancer we found no significant differences (*p* = 0.96) (Figure 5b).

**Figure 6:**
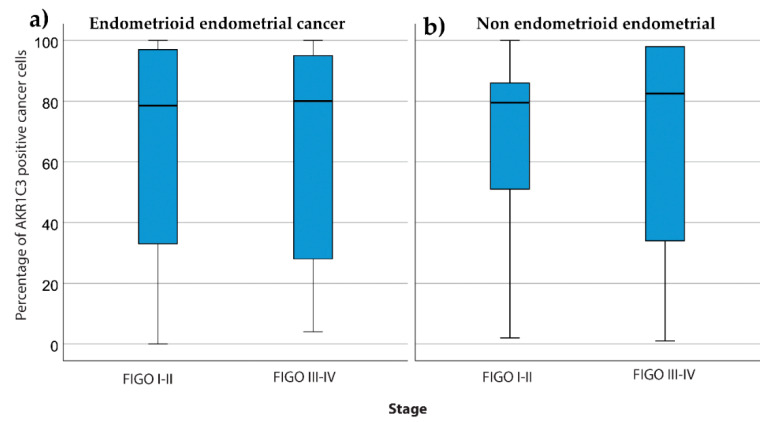
AKR1C3 distribution between patients with different FIGO stages. The box plots show AKR1C3 IHC expression (median, box from 25th to 75th percentiles, whiskers correspond to the 25th percentile minus 1.5 times IQR (interquartile range) and to the 75th percentile plus 1.5 IQR) for patients with different FIGO stages for endometrioid endometrial cancer (**a**) and nonendometrioid endometrial cancer (**b**).

There were no associations between the percentage of AKR1C3 positive cells and FIGO stage for endometrioid endometrial cancer (*p* = 0.83; Mann–Whitney U tests) (Figure 6a) and for nonendometrioid endometrial cancer (*p* = 0.96; Mann–Whitney U tests) (Figure 6b).

Using Mann–Whitney U tests and linear regression analysis, we also evaluated correlations between percentage of AKR1C3 positive cells and the clinical data of the patients with endometrial cancer. In patients with endometrioid and nonendometrioid endometrial cancers, there were no significant correlations between the AKR1C3 expression and patient weight, height, body mass index, menopausal status, parous status, smoking status, use of hormone replacement therapy, presence of lymphovascular invasion, invasion in myometrium or cervix or parametria (Supplementary Materials).

### 3.3. AKR1C3 Levels in Ovarian Cancer and Their Correlations with Survival and Other Clinical Data

In HGSC, as compared to endometrial cancers, there was significantly lower percentage of AKR1C3 positive cells (Figure 2) and there was intratumoral heterogeneity of staining, with relatively sharply delineated areas of positive and negative reactions in the same tissue section of the tumors. IHC reactions were seen for the cytoplasm and nucleus of the cancer cells, and also for the ovarian stromal cells and endothelial cells (Figure 1). The median and mean percentage of AKR1C3 positive cells were 35 and 44.5, respectively.

**Figure 7:**
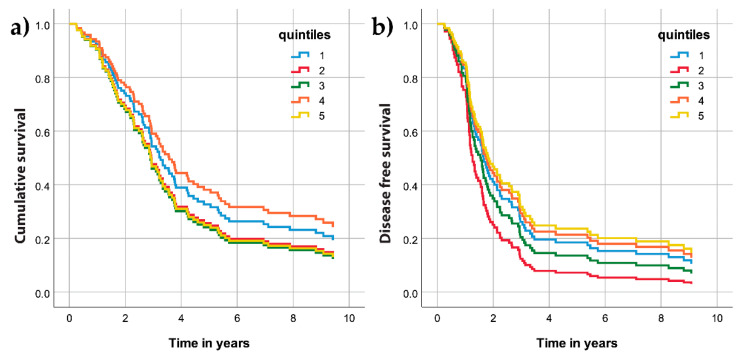
Cumulative survival (**a**) and disease-free survival (**b**) curves for the AKR1C3 quintile groups of patients with high-grade ovarian cancer.

The samples were divided into quintiles according to the percentage of AKR1C3 positive cell distributions, with the following border values: 13, 30, 48, and 85. The computed Cox survival models for the five quintile groups revealed no differences in OS (Figure 7a) or DFS (Figure 7b).

**Figure 8:**
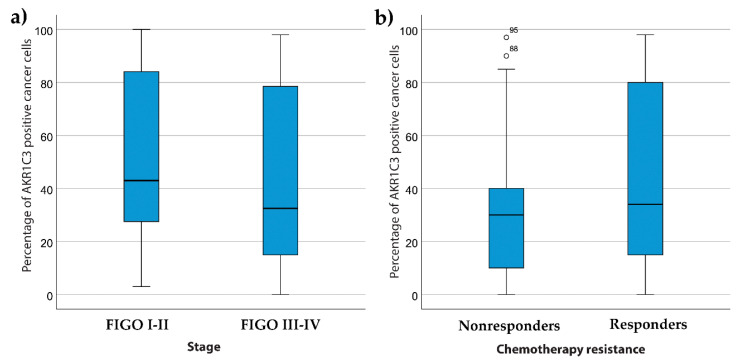
AKR1C3 distributions between patients with high-grade serous ovarian cancer and FIGO stages (**a**) and different response to chemotherapy (**b**). Median values, box from 25th to 75th percentiles, with whiskers that correspond to 25th percentile minus 1.5 times IQR (interquartile range) and to the 75th percentile plus 1.5 IQR are shown.

For patients with ovarian cancer, there were no significant differences between the presence of ascites and the percentage of AKR1C3 positive cells (*p* = 0.23; Mann–Whitney U tests). There were also no significant differences between the different FIGO stages of HGSC (*p* = 0.14; Kruskal–Wallis tests) (Figure 8a).

Next, we looked for correlations between the AKR1C3 expression of the patients with HGSC and their responses to chemotherapy. Similar to previous studies [23], the patients were divided into two groups according to their responses to chemotherapy: (a) non-responders (patients who did not achieve six month DFS); and (b) responders (patients with DFS of at least six months). Here there were no significant differences for the AKR1C3 expression between the responders and nonresponders (*p* = 0.50; Mann–Whitney U tests) (Figure 8b).

## 4. Discussion

This is the first study that has evaluated the expression of AKR1C3 in a large number of patients with endometrial (*n* = 123) and ovarian (*n* = 99) cancers, with the main focus on endometrioid endometrial cancer and HGSC. AKR1C3 levels were evaluated using IHC, and correlations of AKR1C3 with clinicopathological data were studied.

### 4.1. AKR1C3 in Endometrial Cancer

To date, there have only been a few studies that have investigated AKR1C3 levels in endometrial tissue, and endometrial and ovarian cancers. In endometrial tissue, AKR1C3 expression has been reported across the menstrual cycle at the mRNA and protein levels [24,25,26,27]. Significantly increased mRNA levels were shown for the early secretory phase compared to the mid and late secretory phases, the menstrual phase, and the proliferative phase [25]. AKR1C3 immunostaining was observed in glandular and luminal epithelia and in endothelium [25]. In endometrial cancer, increased AKR1C3 mRNA levels have been demonstrated in the model cell lines Ishikawa and HEC-1A versus the control HIEEC cell line [24]. In tissue samples, we previously reported no significant differences in AKR1C3 mRNA levels between endometrial cancer and adjacent control endometrium tissues [28,29], although we observed increased and decreased expression in individual patients [29]. At the protein level, Zakharov et al. (2010) reported decreased AKR1C3 IHC scores in tissues of endometrioid endometrial cancer compared to control endometrium [30]. However, they compared AKR1C3 levels in a very limited number of samples: only three cases of normal proliferative endometrium, eight cases of hyperplastic endometrium with or without atypia, and 12 cases of primary endometrioid endometrial carcinoma grades 2 and 3 [29,30]. Our current data are consistent with this study, as we demonstrated significantly lower AKR1C3 expression in epithelial cells of 61 endometrioid and 11 nonendometrioid endometrial cancer specimens, compared with the adjacent nonneoplastic endometrium from the same patient. AKR1C3 immunoreactivity was seen in endometrial glands, as also reported by Catalano et al. [25].

To date, correlations between AKR1C3 and clinicopathological data and patient survival had not yet been investigated in endometrial and ovarian cancers. The present study revealed that AKR1C3 expression does not significantly correlate with the clinicopathological data. Height, body weight, body mass index, parous status, menopausal status, use of peroral hormone replacement therapy, smoking status, cervical stroma invasion, parametrial invasion, lymphovascular invasion, and lymph node metastases were not significantly correlated with AKR1C3 levels in endometrioid and nonendometrioid endometrial cancers. There were also no significant differences in AKR1C3 levels between different FIGO stages in endometrial cancer. The AKR1C3 IHC reaction was significantly stronger in adjacent nonneoplastic endometrium compared to endometrioid endometrial cancer, and decrease or loss of expression of AKR1C3 in endometrioid endometrial cancer correlated with worse cumulative and disease specific survival. These data suggest that AKR1C3 has a protective role in pathogenesis of endometrioid endometrial cancers, and might be used as an independent prognostic biomarker for endometrioid endometrial cancers. If validated in multicenter studies, AKR1C3 might allow preoperative separation between high-risk endometrial cancer patients (in need of extensive radical surgical treatment and pelvic lymph node dissection) from endometrial cancer patients with good prognoses (in need of radical surgical treatment and sentinel lymph node dissection, with no need for pelvic lymph node dissection), and might thus contribute to tailored personalized treatments, and less postoperative complications.

### 4.2. AKR1C3 in Ovarian Cancer

In ovarian cancer, the levels of AKR1C3 have been evaluated only in model cell lines (e.g., SKOV3, PEO1 cells) and primary cell cultures and tissue samples at the mRNA level, and in a very limited number of paraffin sections of ovarian tissue at the protein level. These studies have reported AKR1C3 expression in cancer cells, and have also described the role of AKR1C3 in steroid hormone metabolism [31,32]. The present study confirms the expression of AKR1C3 in HGSC, with immunoreactivity seen in cancer cells, ovarian stroma, and endothelium.

The present study shows that in HGSC the AKR1C3 levels also do not correlate with any of the clinicopathological data, including the FIGO stage. The high mortality associated with HGSC is partly due to lack of effective screening methods, lack of efficient treatment strategies, and development of resistance to chemotherapy [33]. The mechanisms underlying the development of chemoresistance have been intensively studied in different cancers, although they remain not fully understood [34,35]. The possible mechanisms of resistance include altered cellular transport of the drugs, repair of DNA lesions, increased antioxidant production and detoxification of ROS, reduced apoptosis [36], and metabolism of chemotherapeutics to their less potent metabolites [15,20]. Although the AKR1C enzymes can inactivate cellular stress caused by chemotherapeutics, they can also metabolize several chemotherapeutics [19] and have been associated with chemoresistance of ovarian cancers. In our patients with HGSC, AKR1C3 levels were not significantly correlated with chemotherapy responses or survival. These data indicate that AKR1C3 has no role in proliferation of HGSC and resistance to chemotherapeutics. The other AKR1C enzymes probably have more important roles in chemoresistance than AKR1C3, such as, in particular, AKR1C1 and AKR1C2.

### 4.3. Importance of AKR1C3 in Endometrial and Ovarian Cancers

When we examined publicly available data (i.e., The Cancer Genome Atlas (TCGA) cohort of 1196 patients with endometrioid adenocarcinoma, 489 cases with high-grade serous ovarian cystadenocarcinoma at the cBioPortal for Cancer Genomics; www.cbioportal.org) we did not find any significant differences in survival between patients with mutations, amplifications, or deep deletions of AKR1C3 and patients with unaltered AKR1C3. These publicly available datasets appear to contradict the data here in endometrial cancer. However, gene mutations and amplifications and mRNA levels might not always correspond to protein levels, which might explain these differences. In contrast, in renal and liver cancers, high AKR1C3 expression was significantly associated with unfavorable prognosis [37].

AKR1C3 has an important role in sex hormone metabolism, which potentially influences cancer cells. AKR1C3 has high catalytic efficiency for reduction of androstenedione to testosterone, and it also catalyzes the activation of estrone to the most potent estrogen, estradiol [15]. Recent studies have shown that androgens have important roles in endometrial and ovarian cancers. Studies on cancer cell lines have suggested that androgens inhibit proliferation of endometrial cancer cells while they promote proliferation of ovarian cancer cells [38,39]; androgens are also associated with chemoresistance [40]. Previous studies have shown expression of the androgen receptor (AR) in premenopausal and postmenopausal endometrium and endometrial cancer tissue, with significantly higher expression in postmenopausal endometrium, endometrial hyperplasia, and low-grade endometrial cancer versus proliferative endometrium. AR expression correlated with favorable clinicopathological features and lower proliferation index (Ki67), whereas loss of AR positively correlated with higher tumor grade, higher FIGO stage, and deep myometrial invasion [39,41,42]. These data imply that androgens that act via the AR can have anti-proliferative effects and can serve as prognostic indicators for DFS. As AKR1C3 catalyzes formation of the potent androgen which can activate androgen receptor and can thus inhibit proliferation this supports the hypothesis of the anti-proliferative role of AKR1C3 in endometrial cancer. However, further studies in model cell lines are needed to confirm this hypothesis.

Androgen receptors are also expressed in normal ovaries, 40% of benign ovarian epithelial neoplasms, and 64% of ovarian malignancies (serous, endometrioid and mucinous carcinomas, granulosa cell tumors) [40], which supports a direct role of androgens in ovarian pathophysiology. Androgen signaling can lead to increased cell proliferation and reduced apoptosis, as shown for testosterone and dihydrotestosterone in normal ovarian surface epithelium, as well as in ovarian cancer cell lines [40]. Importantly, IHC has revealed that AR levels decline significantly after chemotherapy [43].

The potential protective effects of androgens in endometrial cancer might help to explain our results here, where higher AKR1C3 levels responsible for formation of active androgen, were associated with better cumulative and disease free survival of patients with endometrioid endometrial cancer. However, to better understand the roles of AKR1C3 in endometrioid endometrial carcinoma and in HGSC, other functions of this enzyme also need to be taken into account, which certainly warrants further studies. To this end, the present study contributes to the global understanding of AKR1C3 functions in cancer.

## 5. Conclusions

In summary, using a large number of samples, we have demonstrated heterogeneous AKR1C3 levels in endometrial and ovarian cancer, with higher expression in endometrial cancer. For endometrioid endometrial cancer, patients with percentages of AKR1C3 positive cells above the median of 79% showed better cumulative survival compared with all of the other patients. In HGSC, AKR1C3 levels did not correlate with survival or response to chemotherapy. These data demonstrate the potential use of AKR1C3 as an independent prognostic biomarker in endometrioid endometrial cancer. Clinically, this might mean that levels of AKR1C3 in endometrioid endometrial cancer can be determined preoperatively on diagnostic tissue samples to allow prediction of the cancer behavior and stratification of the patients for more personalized treatments. This might contribute to less invasive treatments, by limiting pelvic lymph node dissection to only those patients with lower AKR1C3 levels, and higher death risk. However, these findings still require multicenter validation on a larger group of patients with endometrioid endometrial cancer.

## Figures and Tables

**Table 1:** Clinical and histopathological data for the patients with endometrial cancer.

Characteristic	Detail	Datum
Age (y)	Mean ± SD	63.5 ± 11.0
Weight (kg)	Mean ± SD	82.1 ± 18.2
Height (cm)	Mean ± SD	161.9 ± 5.6
Body mass index (kg/m^2^)	Mean ± SD	31.4 ± 7.2
Menopausal status (*N* = 118)^a^ (*n* (%))	Postmenopausal	102 (86.4)
Histological type (*N* = 123) (*n* (%))	Endometrioid	104 (84.6)
	Serous	12 (9.8)
	Dedifferentiated	3 (2.4)
	Carcinosarcoma	2 (1.6)
	Clear cell	1 (0.8)
	Mixed carcinoma	1 (0.8)
Histological grade (*N* = 104) (*n* (%))	G1	67 (64.4)
	G2	25 (24.0)
	G3	12 (11.5)
Myometrial invasion (*n* (%))	<50%	90 (73.1)
	≥50%	33 (26.8)
Lymphovascular invasion (*n* (%))		34 (27.6)
FIGO stage (*N* = 117) ^a^ (*n* (%))	I–II	104 (84.6)
	III–IV	13 (10.6)
Follow-up (y)	Range	0.4–17.6
	Median	7.6
5-year survival (*n* (%))		107 (87.0)

^a^ Cases with missing data: five for menopausal status and six for FIGO stage. N, number of patients; SD, standard deviation.

**Table 2:** Clinical and histopathological data of HGSC patients.

Characteristic	Detail	Datum
Age (y)	Mean ± SD	61.5 ± 11.4
Ascites (*n* (%))		54 (54.5)
Chemotherapy with reported follow-up (*N* = 71) ^a^ (*n* (%))	Responders (at least 6 months DFS)	53 (74.6)
	Non-responders (6 months DFS was not achieved)	18 (25.4)
FIGO stage (*N* = 99)	I–II	19
	III–IV	80
Follow-up (y)	Range	0.3–11.3
	Median	3.1
5-year survival rate ^a^ (*n* (%))		29 (29.3)

^a^ Cases with missing data: 2 with other malignant disease, 6 did not receive chemotherapy, 15 had no follow-up data after chemotherapy, and 5 had no data about treatment. N, number of patients; SD, standard deviation; DFS, disease-free survival.

## Supplementary Materials

The following are available online at https://www.mdpi.com/2077-0383/9/12/4105/s1, Figure S1. Representative immunohistochemistry for AKR1C3 in samples of non-endometrioid endometrial cancer; Figure S2. Cox model showing relationship between hazard ratio for death and AKR1C3 expression in endometrial cancer; Figure S3. Cox model showing relationship between hazard ratio for death and AKR1C3 expression in ovarian cancer; Figure S4. Box plot showing AKR1C3 expression and lymphovascular invasion; Figure S5. Box plot showing AKR1C3 expression and myometrial invasion; Figure S6. Box plot showing AKR1C3 expression and invasion in the cervix; Figure S7. Box plot showing AKR1C3 expression and invasion in the parametria; Figure S8. Box plot showing AKR1C3 expression and menopausal status; Figure S9. Box plot showing AKR1C3 expression and parous status; Figure S10. Box plot showing AKR1C3 expression and smoking status; Figure S11. Box plot showing AKR1C3 expression and use of hormone replacement; Figure S12. Box plot showing the AKR1C3 distribution in patients with ovarian cancer without or with clinical signs of ascites; Table S1. Antibody description and validation.
