# Supplementary material for: AKR1C3 Is Associated with Better Survival of Patients with Endometrial Carcinomas
Source: J Clin Med. 2020 Dec 19;9(12):4105. doi: 10.3390/jcm9124105 (PMC7766127; doi:10.3390/jcm9124105)
Supplement: Supplementary file 1 [file jcm-09-04105-s001.pdf]

Supplementary Data

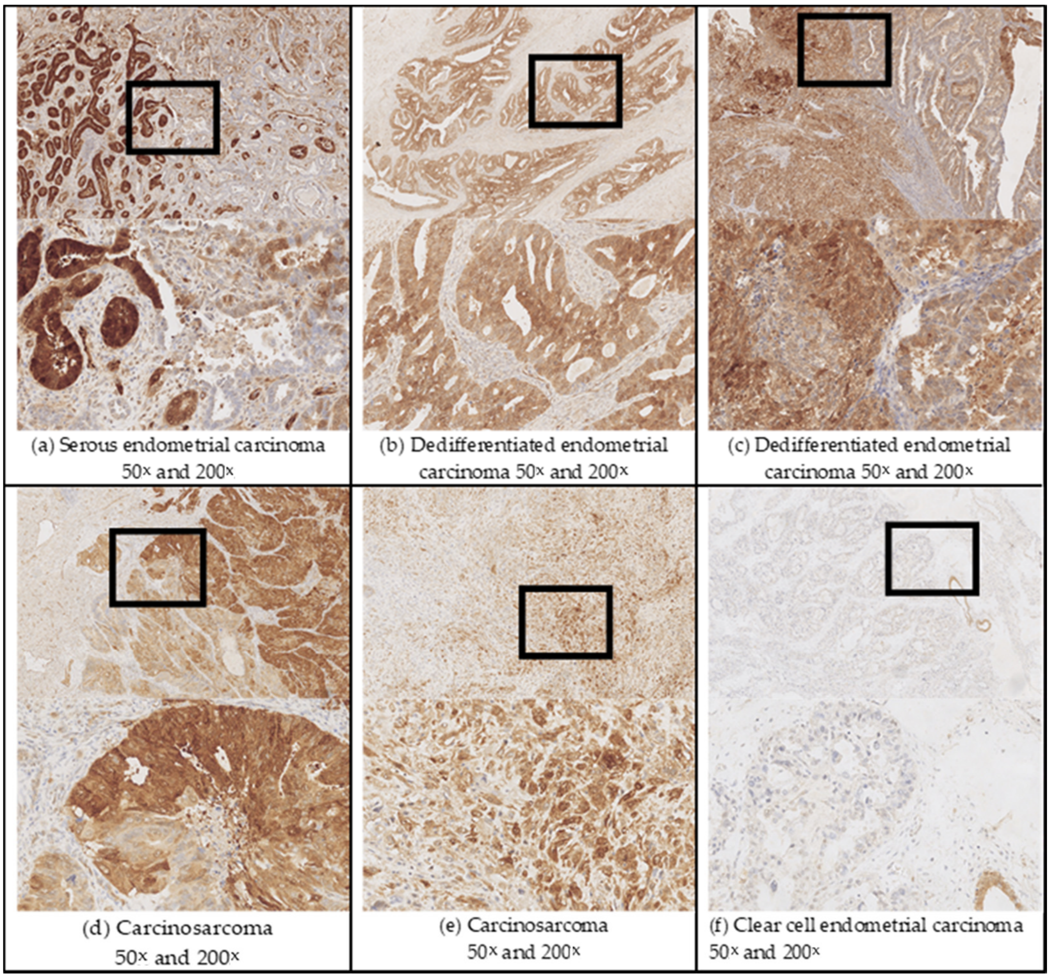

Supplementary Figure S1. Representative IHC for AKR1C3 in samples of non-endometrioid endometrial cancer. Upper half of the picture shows samples at 50x magnification. Black fields represent area, which is shown below at 200x magnification. We observed heterogeneity of staining in serous endometrial cancer and carcinosarcoma – there were relatively sharply delineated areas of positive and negative reaction in the same tissue section of the tumor. Some cases of serous endometrial cancer were diffusely strongly positive. In carcinosarcoma both carcinomatous and sarcomatoid component were positive for AKR1C3 staining. In dedifferentiated endometrial carcinoma we also observed positive reaction for AKR1C3 in both components undifferentiated and in endometrioid endometrial cancer G1 component. One case of clear cell endometrial carcinoma was negative for AKR1C3 staining. In all cases with positive reaction IHC reaction was present in cytoplasm and also in nucleus of cancer cells and in endothelial cell and myometrium. AKR1C3: aldo-keto reductase family 1 member C3; IHC: immunohistochemical.

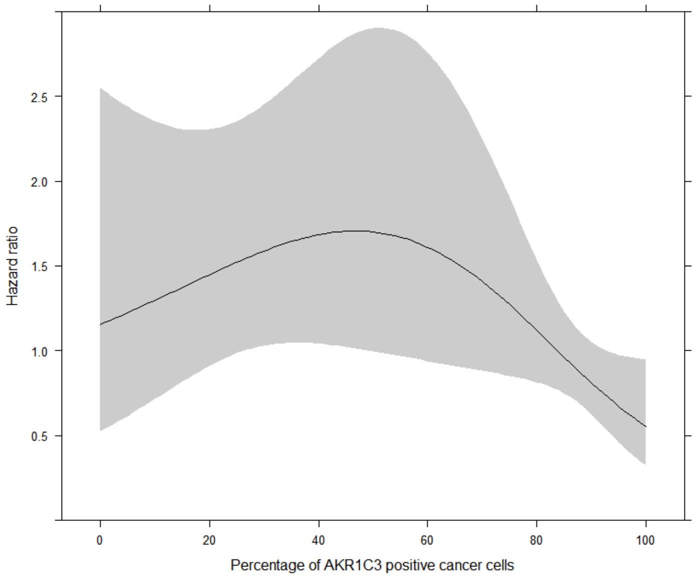

Supplementary Figure S2. Cox model showing relationship between hazard ratio for death and AKR1C3 expression in endometrial cancer. AKR1C3: aldo-keto reductase family 1 member C3.

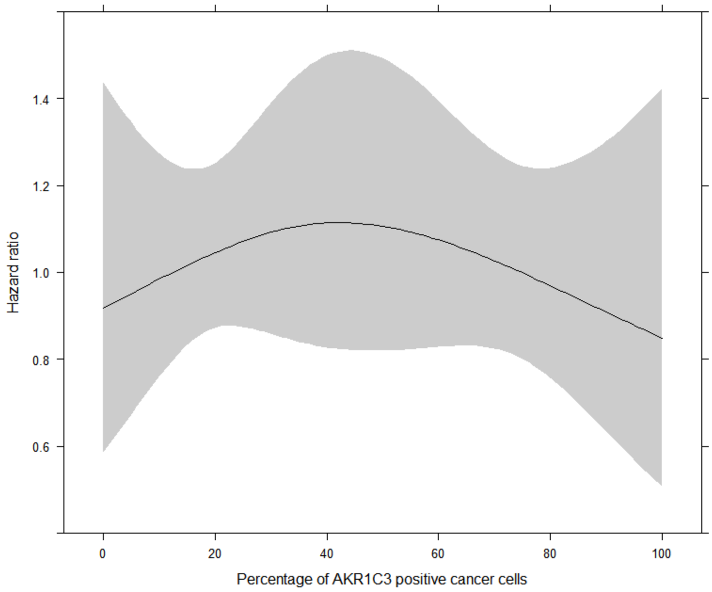

Supplementary Figure S3. Cox model showing relationship between hazard ratio for death and AKR1C3 expression in ovarian cancer.

***Figures S2 and S3** were produced using R 3.6 with the rms and survival libraries. Restricted cubic splines with three knots were used.

References:

Frank E Harrell Jr rms: Regression Modeling Strategies. R package version 5.1-4. https://CRAN.R-project.org/package=rms. **2019.**

Therneau T. A Package for Survival Analysis in S_. version 2.38, <URL: https://CRAN.R-project.org/package=survival. **2015.**

Endometrioid Endometrial Cancer

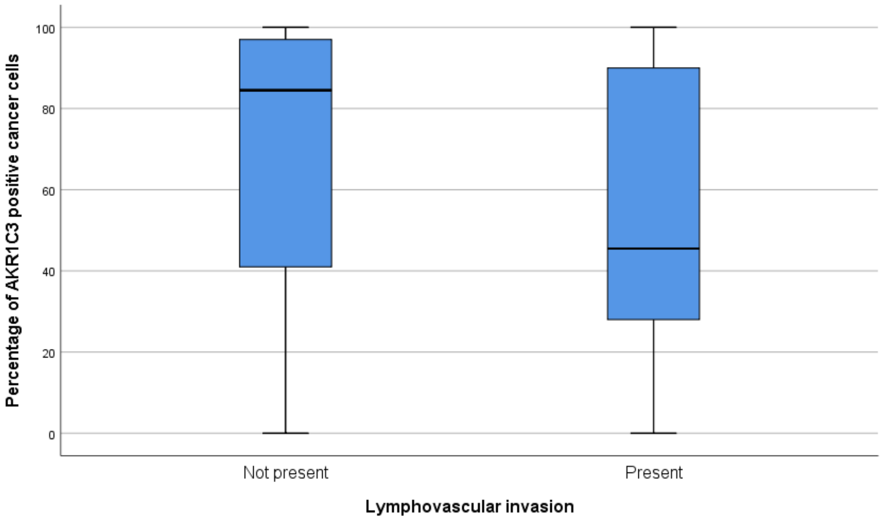

Supplementary Figure S4. Box plot graph showing AKR1C3 expression and lymphovascular invasion. Mann-Whitney U test did not show significantly change, (p = 0.105)

Endometrioid Endometrial Cancer

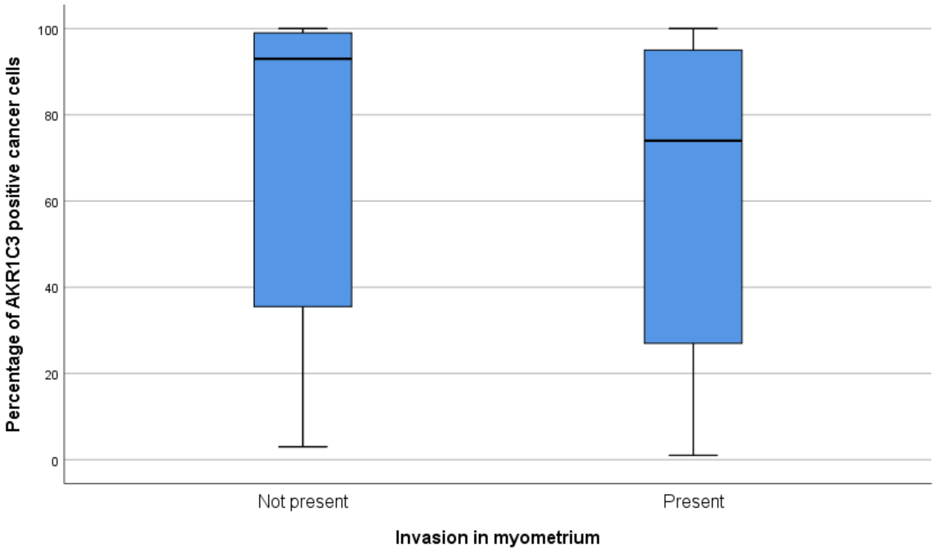

Supplementary Figure S5. Box plot graph showing AKR1C3 expression and myometrial invasion. Mann-Whitney U test did not show significantly change, (p = 0.110). AKR1C3: aldo-keto reductase family 1 member C3.

Endometrioid Endometrial Cancer

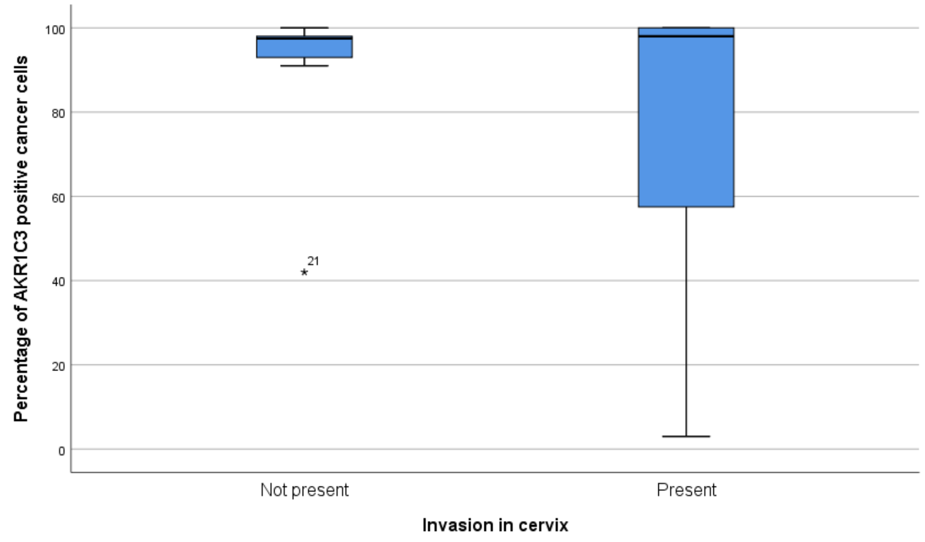

Supplementary Figure S6. Box plot graph showing AKR1C3 expression and invasion in cervix. Mann-Whitney U test did not show significantly change, (p = 0.836).

Endometrioid Endometrial Cancer

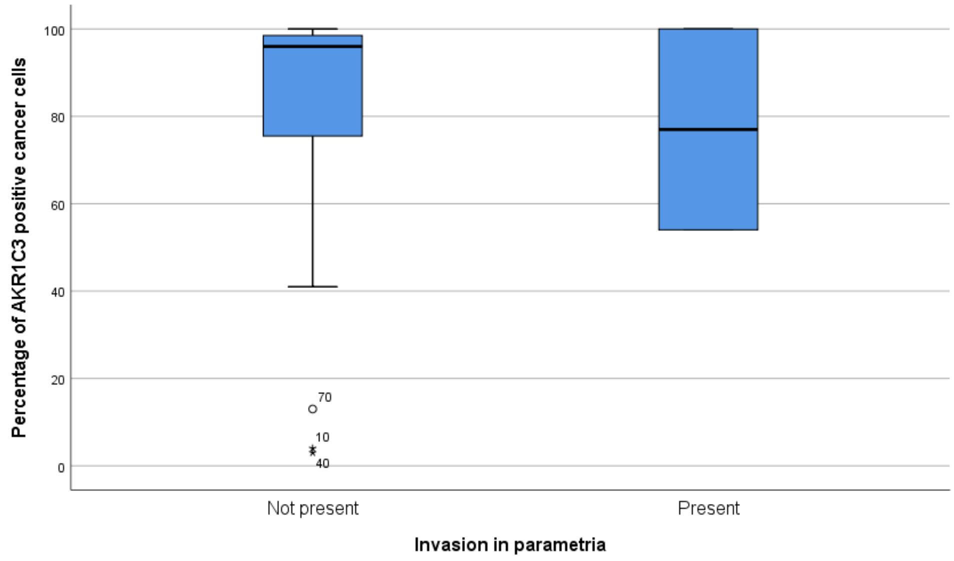

Supplementary Figure S7. Box plot graph showing AKR1C3 expression and invasion in parametria. Mann-Whitney U test did not show significantly change, (p = 0.828).

Endometrioid Endometrial Cancer

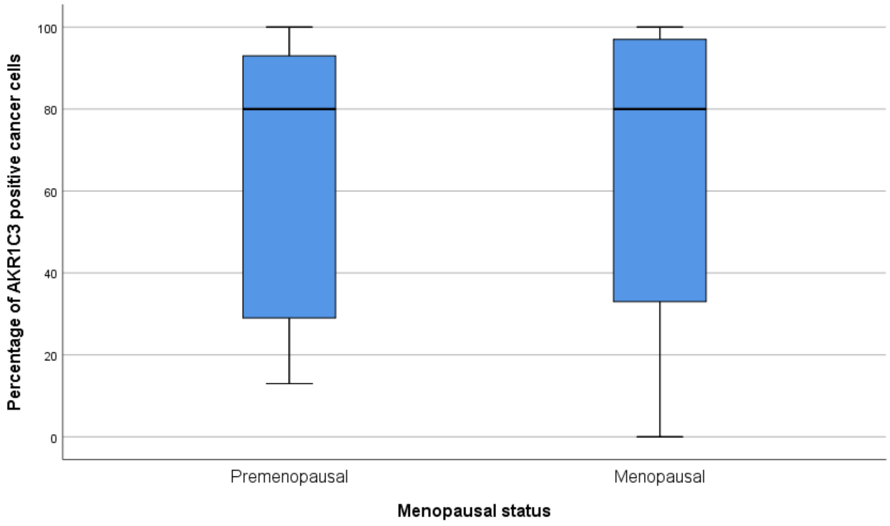

Supplementary Figure S8. Box plot graph showing AKR1C3 expression and menopausal status. Mann-Whitney U test did not show significantly change, (p = 0.930)

Endometrioid Endometrial Cancer

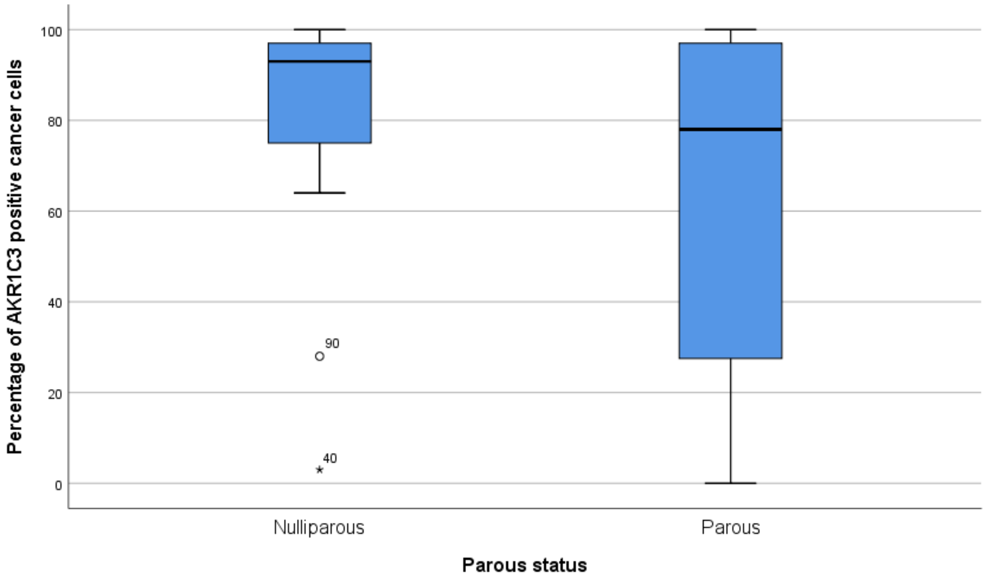

Supplementary Figure S9. Box plot graph showing AKR1C3 expression and parous status. Mann-Whitney U test did not show significantly change, (p = 0.327)

Endometrioid Endometrial Cancer

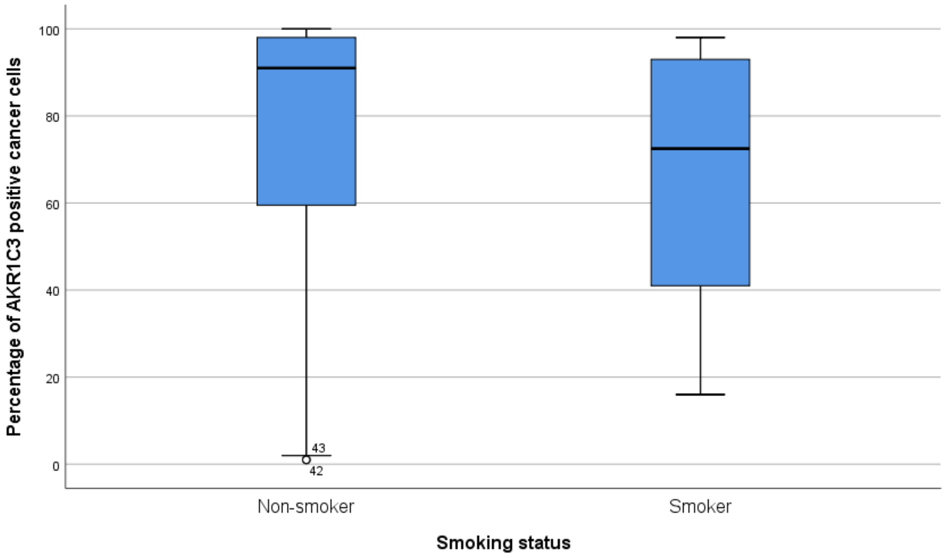

Supplementary Figure S10. Box plot graph showing AKR1C3 expression and smoking status. Mann-Whitney U test did not show significantly change, (p = 0.303).

Endometrioid Endometrial Cancer

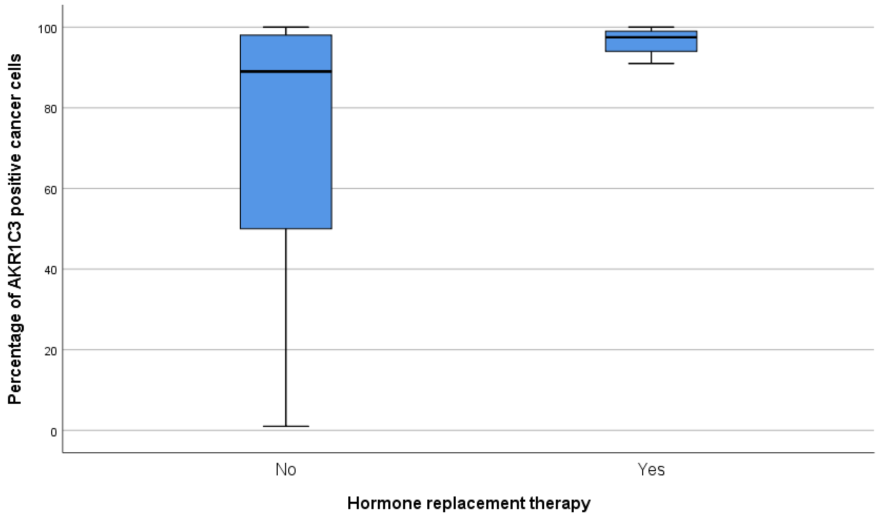

Supplementary Figure S11. Box plot graph showing AKR1C3 expression and use of hormone replacement therapy. Mann-Whitney U test did not show significantly change, (p = 0.152).

High Grade Serous Ovarian Cancer

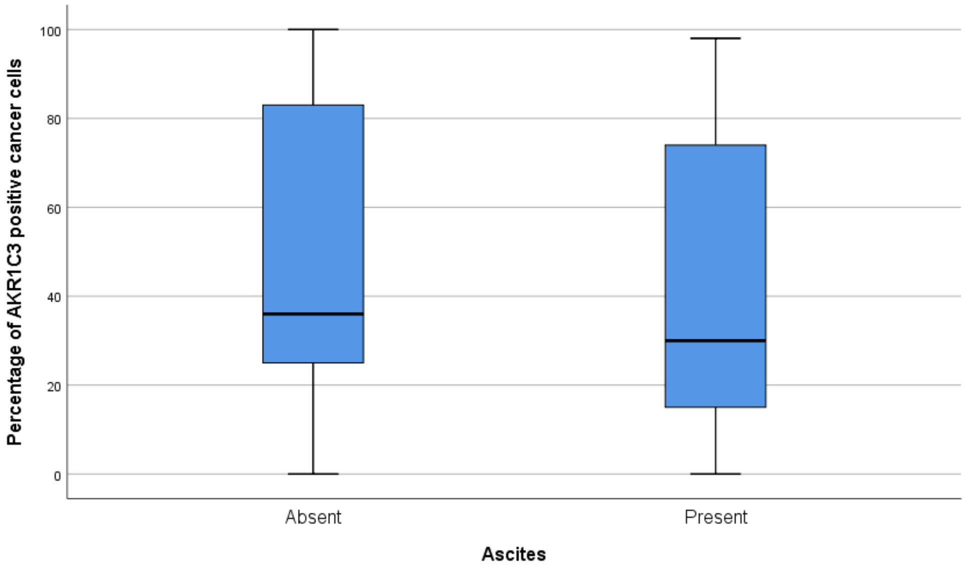

Supplementary Figure S12. The box plot graph showing distribution of AKR1C3 in patients with high-grade serous ovarian cancer with or without clinical signs of ascites. Mann-Whitney U test did not show significantly change, (p = 0.230).

Table S1. Antibody description and validation.

<table>
<tr><th colspan="6">Antibody information</th></tr>
<tr><th>Name of the antibody</th><th>Manufacturer, catalogue number</th><th>Peptide/ protein target</th><th>Antigen sequence</th><th>Species raised, monoclonal, polyclonal, antigen purified</th><th>Dilution used (mass concentration if affinity purified), reagents</th></tr>
<tr><td>Anti-AKR1C3, clone NP6.G6.A6</td><td>Sigma-Aldrich, A6229</td><td>Aldo-keto reductase family 1 member C3</td><td>Whole recombinant protein</td><td>Monoclonal mouse antibodies purified from hybridoma cell culture</td><td>1:400</td></tr>
<tr><th colspan="6">Antibody validation</th></tr>
<tr><td colspan="6">Published validation:
Western blotting with recombinant enzymes AKR1C1–4 confirmed specificity for AKR1C3 only. IHC staining of breast and prostate paraffin-embedded tissue specimen containing normal and cancer tissue, also after pre-absorption with recombinant AKR1C3 [31,32].</td></tr>
<tr><td colspan="6">Our validation:
Positive controls: large intestine, liver.
Negative controls: lungs, heart muscle.</td></tr>
</table>

AKR1C3: aldo-keto reductase family 1 member C3; IHC: immunohistochemical.
